# miR-6734 Up-Regulates p21 Gene Expression and Induces Cell Cycle Arrest and Apoptosis in Colon Cancer Cells

**DOI:** 10.1371/journal.pone.0160961

**Published:** 2016-08-10

**Authors:** Moo Rim Kang, Ki Hwan Park, Jeong-Ook Yang, Chang Woo Lee, Soo Jin Oh, Jieun Yun, Myeong Youl Lee, Sang-Bae Han, Jong Soon Kang

**Affiliations:** 1 Bio-Evaluation Center, Korea Research Institute of Bioscience and Biotechnology, Cheongju, Republic of Korea; 2 College of Pharmacy, Chungbuk National University, Cheongju, Republic of Korea; Universitat des Saarlandes, GERMANY

## Abstract

Recently, microRNAs have been implicated in the regulation of gene expression in terms of both gene silencing and gene activation. Here, we investigated the effects of miR-6734, which has a sequence homology with a specific region of p21^WAF1/CIP1^ (p21) promoter, on cancer cell growth and the mechanisms involved in this effect. miR-6734 up-regulated p21 expression at both mRNA and protein levels and chromatin immunoprecipitation analysis using biotin-labeled miR-6734 confirmed the association of miR-6734 with p21 promoter. Moreover, miR-6734 inhibited cancer cell growth and induced cell cycle arrest and apoptosis in HCT-116 cells, which was abolished by knockdown of p21. The phosphorylation of Rb and the cleavage of caspase 3 and PARP were suppressed by miR-6734 transfection in HCT-116 cells and these effects were also reversed by p21 knockdown. In addition, miR-6734 transfection caused prolonged induction of p21 gene and modification of histones in p21 promoter, which are typical aspects of a phenomenon referred to as RNA activation (RNAa). Collectively, our results demonstrated that miR-6734 inhibits the growth of colon cancer cells by up-regulating p21 gene expression and subsequent induction of cell cycle arrest and apoptosis, suggesting its role as an important endogenous regulator of cancer cell proliferation and survival.

## Introduction

Small RNA molecules, such as short interfering RNA (siRNA) and microRNA (miRNA), have been known as important regulators of gene expression. These small RNA molecules have been typically known to repress gene expression by binding to mRNA and consequently leading to degradation of mRNA or inhibition of translation [1,2]. However, lines of evidence suggested that small non-coding double strand RNA (dsRNA) could induce sequence-specific transcriptional gene activation by targeting specific region in a cognate gene promoter [3,4]. This phenomenon has been termed as RNA-induced gene activation (RNAa) and the gene-activating dsRNA was termed as a small activating RNA (saRNA) [3,5]. RNAa was known to have unique kinetics and the induction of gene expression by saRNA prolongs even after cell passage and lasts for nearly 2 weeks, which is different from the kinetics of siRNA-mediated gene silencing [5]. In addition, it has been reported that saRNAs induces histone modification at promoter region and recruits RNA polymerase II (RNAP II) [4].

miRNAs are non-coding small RNAs composed of 20~30 nucleotides and a number of reports showed that miRNAs might play important roles in various biological processes, including cell proliferation, apoptosis and differentiation [6]. Recently, it has been reported that miRNAs can activate transcription, similar to saRNA, by binding to promoter of various genes [7–9]. Place and coworkers reported that miR-373, which has a sequence homology with E-cadherin promoter, induced E-cadherin gene expression by targeting its promoter [7]. In addition, Huang and coworkers also showed that an overexpression of miR-744 and miR-1186 induced cyclin B1 expression and enhanced cell proliferation, which was accompanied by increased RNAP II recruitment and histone H3 lysine 4 tri-methylation at promoter region [10]. Therefore, these results suggest that promoter-targeting miRNAs may induce transcriptional gene activation in a manner similar to saRNA.

Previous studies showed that p21^WAF1/CIP1^ (p21) promoter-targeting saRNA, dsP21-322, possesses *in vitro* antigrowth activity in various cancer cells and *in vivo* antitumor activity in orthotopic model of bladder cancer [11–13]. Using *in silico* analysis, we found that miR-6734 has a sequence similarity with dsP21-322 and there is a highly-complementary site for miR-6734 in p21 promoter. Therefore, we investigated the effects of miR-6734 on p21 expression and cell proliferation in HCT-116 colon cancer cells. We also examined the effect of miR-6734 on cell cycle distribution and apoptosis induction in HCT-116 cells. Our results suggest that miR-6734 is a novel regulator of p21 gene expression and suppresses cell proliferation and survival in colon cancer cells.

## Materials and Methods

### Cell culture and transfection

The cell lines HCT-116 (ATCC CCL-247), PC3 (ATCC CRL-1435), NUGC-3 (JCRB0822), Caski (ATCC CRL-1550) and MDA-MB-231 (ATCC HTB-26) were cultured in RPMI 1640 medium (Gibco BRL; Grand Island, NY, USA) supplemented with 10% fetal bovine serum (Hyclone; Logan, UT, USA), 2 mM L-glutamine, 100 U/ml penicillin and 100 μg/ml streptomycin. Cells were maintained at 37°C in a humidified atmosphere containing 5% CO_2_. miR-6734 mimic, miR-6734-5P inhibitor, biotin-linked miR-6734, dsP21-322, siP21, and control dsRNA (dsCon) were chemically synthesized and supplied by Bioneer (Daejeon, Republic of Korea). All dsRNA sequences are listed in S1 Table. dsRNA or miRNA was transfected using Lipofectamine RNAiMax reagent (Invitrogen Life Technologies; Carlsbad, CA, USA).

### RNA isolation and quantification of mRNA expression

Cells were plated at 1 x 10^5^ cells/well in 6-well plates, incubated overnight, and transfected with various concentration of dsRNA or miRNA. Total cellular RNA was extracted using the RNeasy Mini Kit (Qiagen; Venlo, Netherlands) with RNase-Free DNase Set (Qiagen; Venlo, Netherlands) following the manufacturer’s instructions. RNA (1 μg) was used for cDNA synthesis using the PrimeScript 1^st^ strand cDNA synthesis kit (Takara; Shiga, Japan). The resulted cDNA was amplified both by qPCR in conjunction with Power SYBR Green PCR Master Mix (Invitrogen Life Technologies; Carlsbad, CA, USA) and RT-PCR. In qPCR, samples were amplified by 40 cycles of denaturation (95°C for 15 s) and amplification (60°C for 1 min) using ABI 7500 Sequence Detection System (Applied Biosystems; Carlsbad, CA, USA). RT-PCR amplification consisted of an initial denaturation step (95°C for 3 min), 30 cycles of denaturation (95°C for 30 s), annealing (58°C for 30 s), and extension (72°C for 60 s) followed by a final incubation at 72°C for 5 min. The amount of each cDNA was normalized by the amount of GAPDH. All primer sequences are listed in S1 Table and the concentration of primers used was 10 pmol/L.

### Cell proliferation assay

Cells were plated at 4 x 10^3^ cells/well in 96-well plates, incubated overnight, and transfected with various concentration of dsRNA or miRNA. Cell growth was measured at 4 time points from day 1 to day 4 following transfection. Cell proliferation assays were performed using a Cell Proliferation Kit II (Roche Applied Science; Mannheim, Germany) according to the manufacturer's instructions. Briefly, XTT (sodium 3’-[1-(phenylaminocarbonyl)-3,4-tetrazolium]-bis(4-methoxy-6-nitro)benzene sulfonic acid hydrate) labeling mixture was added to the cultures and incubated for 2 h at 37°C. Absorbance was measured at 490 nm with a reference wavelength at 650 nm.

### Clonogenic survival assay

Cells were transfected in 6-well plates at 50% density. The following day, the cells were seeded in a 6-well plate at a cell density of 2,500 cells per well and were maintained for 10 days with the culture medium changed every 2 days. At day 10, after removing the medium and washing the cells with PBS, the resulted cell colonies were stained with 0.05% crystal violet for 10 min at room temperature and photographed.

### Cell cycle analysis

Cells were plated at 1 x 10^5^ cells/well in 6-well plates, incubated overnight, and transfected with various concentration of dsRNA or miRNA. Cells were harvested and centrifuged at 1,000xg for 5 min and washed with ice-cold PBS. The cells were resuspended with 100 μl of ice-cold 70% ethanol and incubated overnight at 4°C. The cells were centrifuged at 3,000 rpm for 1 min and resuspended in 100 μl Krishan Buffer (0.1% sodium citrate, 0.03% Triton X- 100, 0.02mg/mL RNase A, 0.05mg/mL propidium iodide) and incubated for 1 h at 4°C. Then the stained cells were analyzed using a FACSCalibur flow cytometer (BD Biosciences; San Jose, CA, USA). The experiments were repeated at least 3 times and, 10,000 events were analyzed for each sample. The data was analyzed using ModFit LT program (Verity Software House; Maine, ME, USA, http://www.vsh.com/products/mflt/mfFeatures.asp).

### Western blotting analysis

Total protein extracts were prepared by lysing cells in Cell Lysis Buffer (Cell Signaling Technology; Beverly, MA, USA) with protease inhibitor cocktail (Merck Millipore; Billerica, MA, USA) and phosphatase inhibitor (Sigma-Aldrich; St. Louis, MO, USA). Protein concentrations in the lysates were determined using a BCA protein Assay Kit (Pierce Biotechnology; Waltham, MA, USA) according to the manufacturer's instructions. Protein extracts were separated by 8~12% SDS-polyacrylamide gel electrophoresis and transferred to nitrocellulose membranes. The membranes were incubated with primary antibodies against p21 (Cat #: 2947, 1:2,000 dilution), p-Rb (Cat #: 9308, 1:1,000 dilution), caspase-3 (Cat #: 9662, 1:1,000 dilution), PARP (Cat #: 9542, 1:1,000 dilution), cyclin A (Cat #: 4656, 1:1,000 dilution) or GAPDH(Cat #: 2118, 1:2,500 dilution) (Cell Signaling Technology; Beverly, MA, USA). After washing, membranes were probed with horseradish peroxidase-conjugated secondary antibodies (1:5,000 dilution, Cell Signaling Technology; Beverly, MA, USA). Detection was performed using an Immobilon Western Chemiluminescent HRP substrate (Merck Millipore; Billerica, MA, USA).

### Apoptosis assay and caspase 3/7 activity assay

Apoptosis analysis was performed using an Annexin V-FITC Apoptosis Detection Kit II (BD Bioscience; San Jose, CA, USA) according to the manufacturer's instructions. Briefly, cells were plated at 1 x 10^5^ cells/well in 6-well plates, incubated overnight, and transfected with various concentration of dsRNA or miRNA. Cells were harvested and combined with a binding buffer containing Annexin-V-FITC and propidium iodide. Following 15 min incubation in the dark, cells were analyzed by flow cytometry using a FACSCalibur flow cytometer (BD Bioscience; San Jose, CA, USA). The data were analyzed using WinMDI software (The Scripps Research Institute; La Jolla, CA, USA, http://facs.scripps.edu/software.html). The activities of caspases 3/7 were determined via a Caspase-Glo 3/7 assay (Promega; Madison, WI, USA) conducted in accordance with the manufacturer's instructions. In brief, the culture supernatants were collected on 96-well turbid microtiter plates and 50 μl of proluminescent caspase-3/7 substrates were added. After 1 h of incubation at 37°C, luminescence was measured using a Victor Light system (PerkinElmer; Waltham, MA, USA).

### Chromatin immunoprecipitation (ChIP) assay

Chromatin immunoprecipitation (ChIP) was performed as previously described with slight modification [14]. Briefly, transfected cells were cross-linked with 1% formaldehyde for 10 min at room temperature, and the cross-linking reaction was stopped by adding glycine to a final concentration of 0.125 M. DNA was sheared to an average size of ~500 bp using the Vibra cell sonicator (Sonics and Materials inc; Newtown, CT, USA). The sonicated mixture was centrifuged at 14,000 rpm for 15 min at 4°C, and the supernatant was collected. Immunoprecipitation was performed using 4 μg/sample of anti-IgG (Cat #: sc-2768), anti-biotin (Cat #: sc-57636, Santa Cruz Biotechnology, Inc.; Dallas, TX, USA), anti-H2Bac (Cat #: 07–371), anti-H3ac (Cat #: 06–599), and anti-H3m2K9 (Cat #: 07–441, Merck Millipore; Billerica, MA, USA) antibodies. Fresh beads were then added for 2 h and washed. Samples were subsequently treated with Proteinase K and RNase A followed by phenol/chloroform extraction and analyzed by PCR. PCR primers used for ChIP analysis are described in S1 Table.

### Statistical analysis

Results are expressed as the mean ± SD. One-way ANOVA followed by Dunnett's t-test were used for statistical analysis using GraphPad Prism (GraphPad Software; La Jolla, CA, USA). The criteria for statistical significance were set at *p < 0.05, **p < 0.01 and ***p < 0.001.

## Results

### miR-6734 induces p21 gene expression by binding to p21 promoter

**Fig 1:**
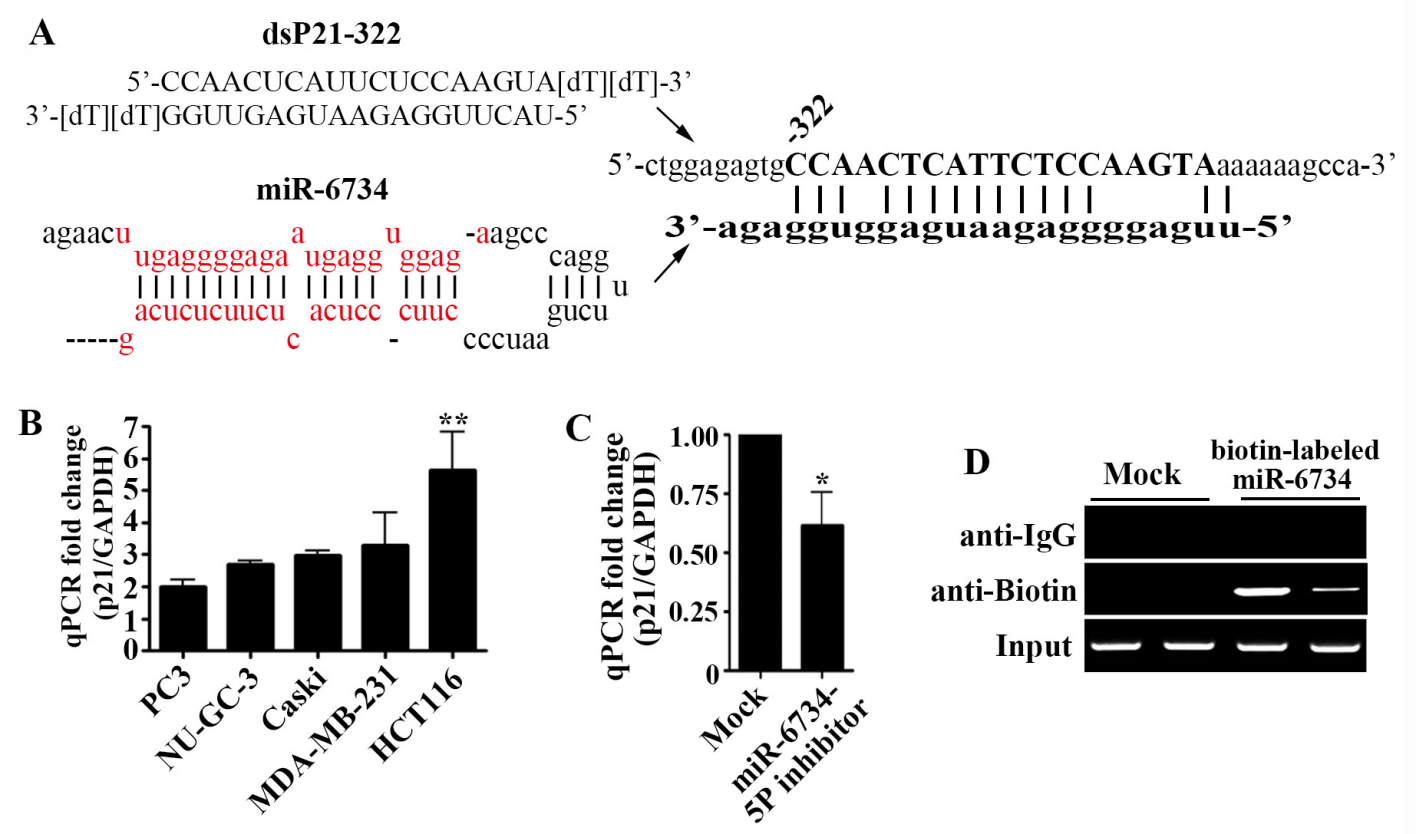
miR-6734 targets p21 promoter and induces p21 gene expression. (A) Sequence of dsP21-322 and miR-6734 target site located at nucleotide -322 relative to the transcriptional start site in p21 promoter. (B) Five human cancer cells were transfected with miR-6734 at 10nmol/L for 72h. The mRNA expression of p21 was analyzed by qPCR. (C) HCT-116 cells were transfected with mock and miR-6734-5P inhibitor at 30 nmol/L for 72 h. The mRNA expression of p21 was analyzed by qPCR. Data are presented as mean ± S.D. of quadruplicate experiments. Statistical significance was analyzed by one-way ANOVA and Dunnett’s t-test (* p < 0.05; ** p < 0.01 versus mock). (D) HCT-116 cells were transfected with biotin-labeled miR-6734 and chromatin immunoprecipitation (ChIP) assays were performed by using antibodies against biotin to pull down associated DNA. The precipitated DNA was amplified by PCR using primer sets specific to miR-6734 target sites (-360/-260) of p21 promoter. Input DNA was amplified as a loading control. DNA pulled down by the anti-IgG antibody was served to identify background amplification. Duplicate samples were analyzed for each treatment.

It was reported that miRNAs can modulate gene expression by targeting promoter regions [7,10]. To identify candidate miRNAs that may be involved in the regulation of p21 gene expression, we performed a scanning analysis in p21 promoter region using the sequence of dsP21-322, a previously reported saRNA known to induce p21 gene expression [3,12,15], as a reference. Among miRNAs included in miRBASE database (www.mirbase.org), miR-6734 was found to be highly complementary to p21 promoter (Fig 1A).

To investigate whether miR-6734 can modulate p21 gene expression, we assessed the effect of miR-6734 on the mRNA expression of p21 gene in various cancer cell lines, including PC3, NUGC-3, Caski, MDA-MB-231 and HCT-116. As shown in Fig 1B, transfection of miR-6734 (10 nmol/L) elicited 2.0, 2.7, 3.0, 3.3, and 5.7-fold induction of p21 mRNA levels in PC3, NUGC-3, Caski, MDA-MB-231 and HCT-116 cells, respectively. To determine the endogenous function of miR-6734 on p21 mRNA expression, we transfected HCT-116 cells with miR-6734-5P inhibitor that targets miR-6734 and blocks its function. Fig 1C shows that miR-6734-5P inhibitor (30 nmole/L) significantly reduced basal level of p21 mRNA expression in HCT-116 cells. To further investigate whether miR-6734 exerts its function by directly binding to p21 promoter, we performed chromatin immunoprecipitation assay using a biotin-labeled miR-6734 in HCT-116 cells. As shown in Fig 1D, biotin-labeled miR-6734 pulled down p21 promoter region which is located at -360 to -260 relative to transcription starting site.

### miR-6734 inhibits cell growth and survival in HCT-116 cells

**Fig 2:**
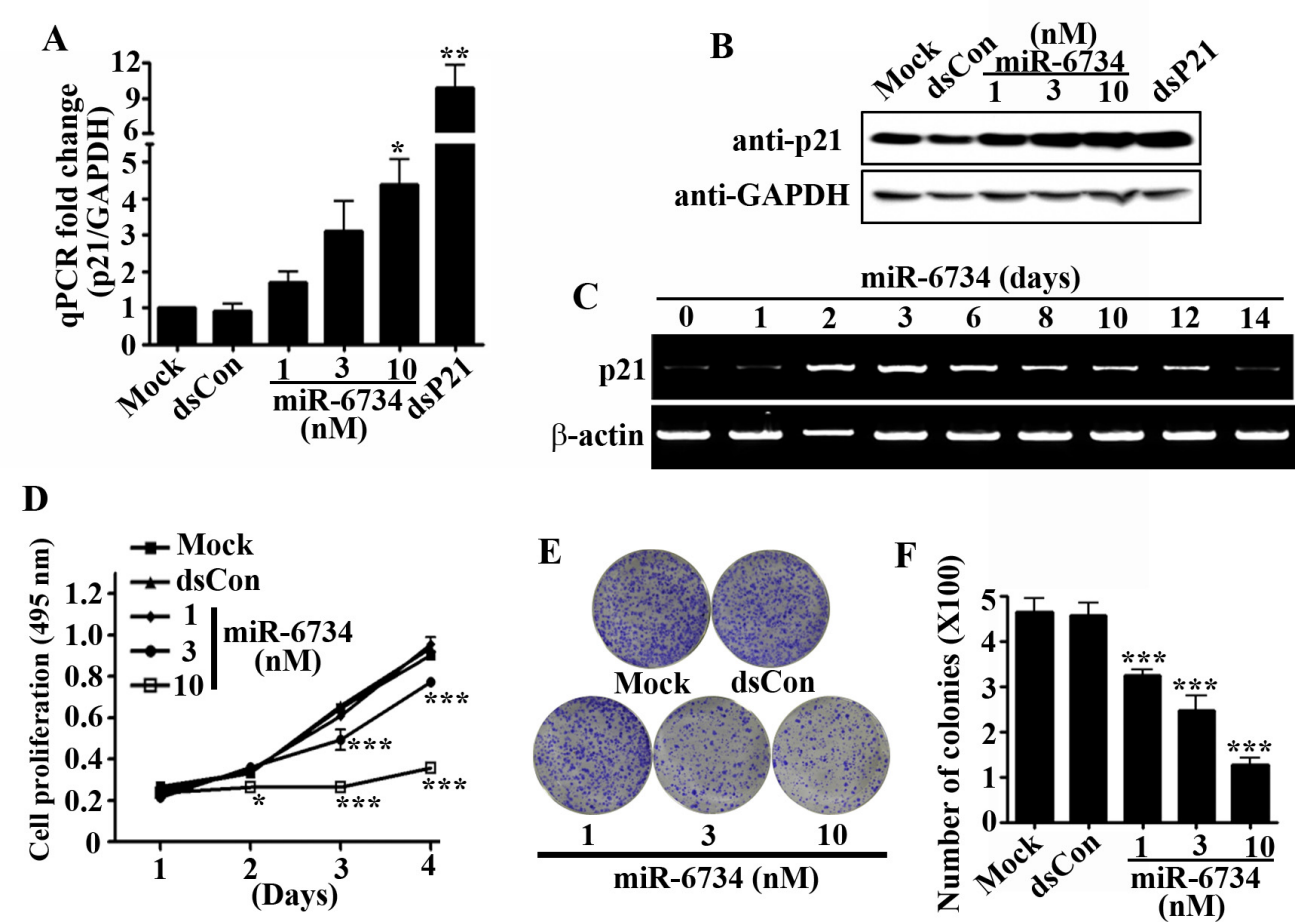
miR-6734 induced p21 expression and inhibits proliferation and survival in HCT-116 colon cancer cells. (A,B) HCT-116 cells were transfected with mock, dsCon and dsP21-322 at 10 nmol/L or the indicated concentrations of miR-6734 for 72 h. The mRNA expression of p21 was determined by qPCR and the protein expression of p21 was determined by Western Immunoblot analysis. (C) Cells were transfeced with 10 nmol/L miR-6734 for the indicated lengths of time. The mRNA expression of p21 and β-actin was assessed by RT-PCR. (D) Cell proliferation was measured from day 1 to 4 after treatment with the indicated concentrations of miR-6734 using the Cell proliferation kit (XTT). (E,F) The treated cells were seeded in 6-well plates at a density of 2,500 cells per well. Colony formation was analyzed on day 10 by staining the cells with crystal violet and the number of colonies was counted. Data are presented as mean ± S.D. of triplicate experiments. Statistical significance was analyzed by one-way ANOVA and Dunnett’s t-test (* p < 0.05; *** p < 0.001 versus mock).

Here, we confirmed the dose-related effects of miR-6734 on mRNA and protein expression of p21 gene in HCT-116 cells. As shown in Fig 2A and 2B, transfection of HCT-116 cells with miR-6734 induced both mRNA and protein expression of p21 in a dose-dependent manner. In addition, dsP21-322 also dramatically induced the expression of p21 gene at both mRNA and protein level (Fig 2A and 2B). Previous studies reported that gene activation by a single transfection of saRNA can last almost 2 weeks [5]. To investigate whether miR-6734 has a charateristic similar to saRNA, time-course of p21 gene expression was evaluated by RT-PCR. As shown in Fig 2C, the up-regulation of p21 by a single transfection of miR-6734 was observed at day 2 and lasted until day 12.

The induction of p21 gene expression by dsP21-322 was shown to cause cell growth inhibition in various cells [3,12,15]. Therefore, we investigated the effect of miR-6734 on cell proliferation in HCT-116 cells. As illustrated in Fig 2D, miR-6734 inhibited the proliferation of HCT-116 cells in a dose-related manner. To further evaluate the effect of miR-6734 on cell survival, colony formation assay was performed. As shown in Fig 2E and 2F, mock and dsCon-transfected HCT-116 cells formed numerous colonies after 10 days, whereas the colony formation of cells transfected with miR-6734 was reduced significantly in dose-dependent manner.

### miR-6734 induces cell cycle arrest and apoptosis in HCT-116 cells

**Fig 3:**
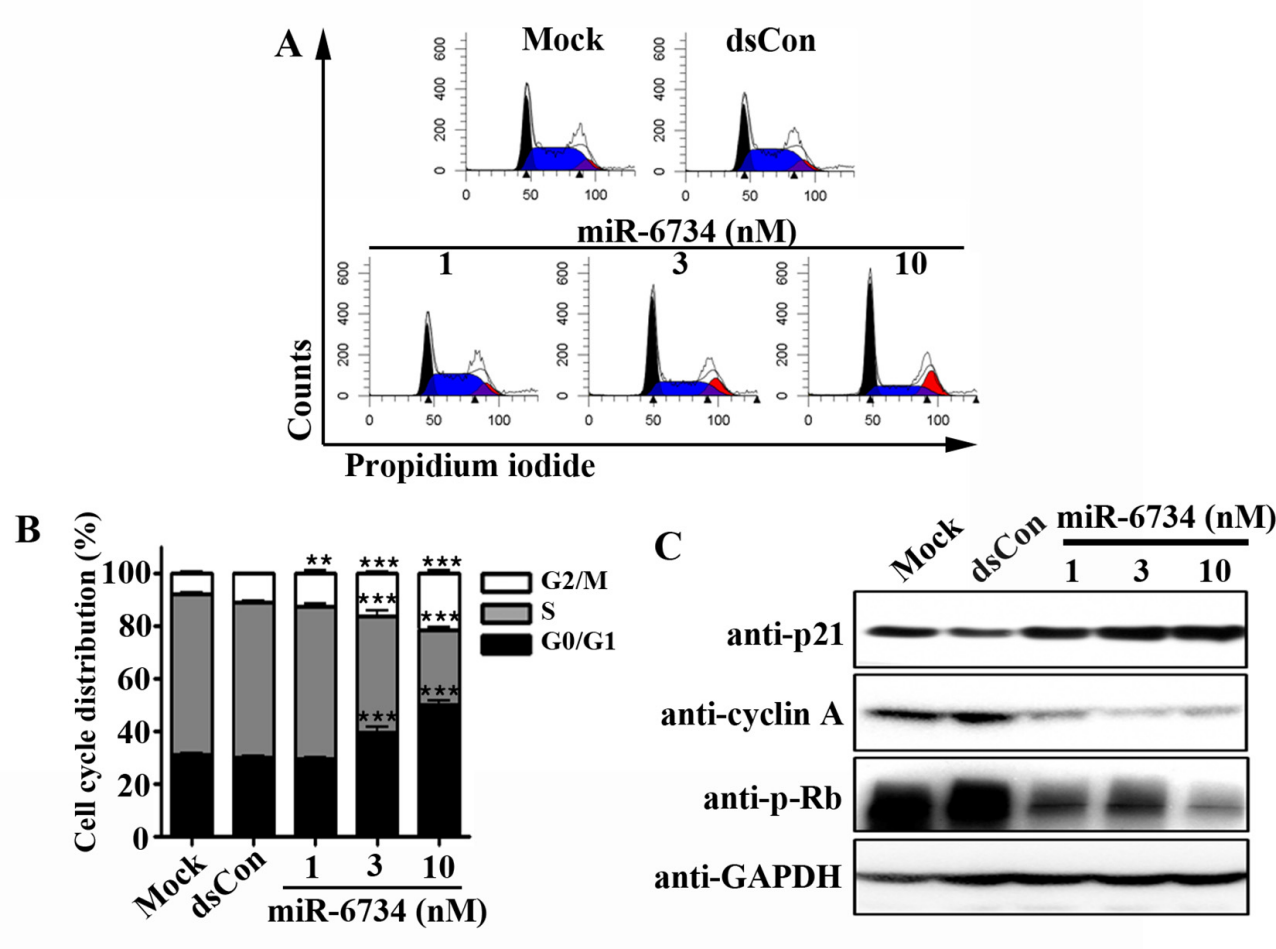
miR-6734 induces cell cycle arrest in HCT-116 cells. HCT-116 cells were transfected with mock, dsCon or the indicated concentrations of miR-6734 for 48 h. (A) Cell cycle distribution was examined by flow cytometry. (B) The percentage of cells in G0/G1, S, and G2/M phases were calculated using the ModFit program. Data are presented as mean ± S.D. of triplicate experiments. Statistical significance was analyzed by one-way ANOVA and Dunnett’s t-test (** p < 0.01; *** p < 0.001 versus mock). (C) Protein levels of p21, cyclin-A, p-Rb and GAPDH in the total cell lysates were determined by Western Immunoblot analysis.

**Fig 4:**
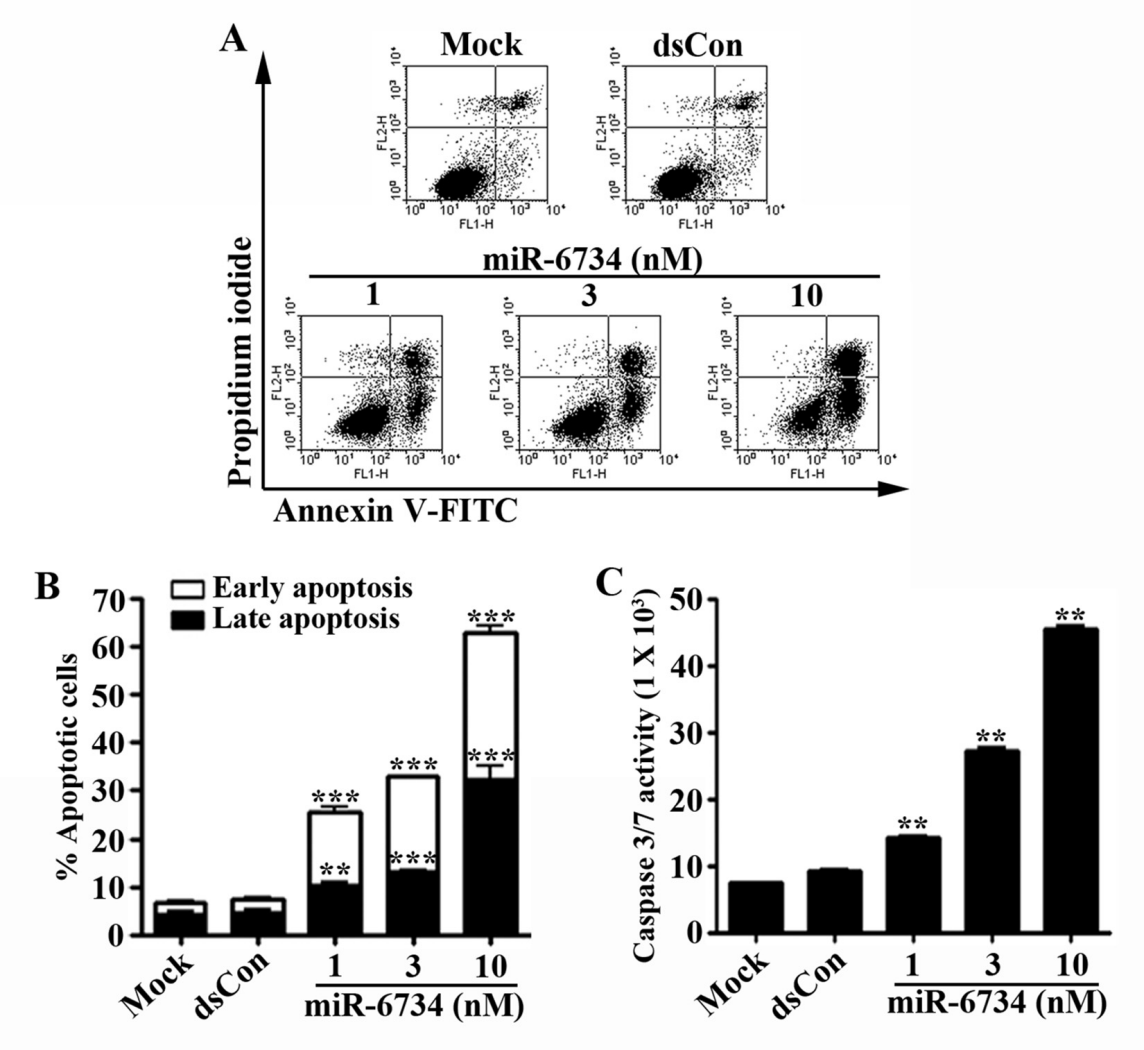
miR-6734 induces apoptosis in HCT-116 cells. HCT-116 cells were treated with mock, dsCon or the indicated concentrations of miR-6734 for 72 h. (A) Transfected cells were stained with propidium iodide and annexin V-FITC and apoptotic cells were measured by flow cytometry. (B) The percentage of early and late apoptotic cells were calculated using the WinMDI program. (C) Culture supernatants were collected, and the activity of caspase-3/7 was determined via caspase-Glo 3/7 assay kit. Data are presented as mean ± S.D. of triplicate experiments. Statistical significance was analyzed by one-way ANOVA and Dunnett’s t-test (** p < 0.01; *** p < 0.001 versus mock).

To determine whether p21 induction by miR-6734 have an effect on cell cycle distribution, DNA content was analyzed by flow cytometry in cells transfected with miR-6734. Fig 3A and 3B shows that the accumulation of cells at G0/G1 and G2/M phase and loss of cells at S phase were detected after transfection of HCT-116 cells with miR-6734. To further confirm, we also analyzed the effect of miR-6734 on cyclin A and retinoblastoma protein (Rb), which are well-known to regulate cell. As shown in Fig 3C, the level of cyclin A and the phosphorylation of Rb was significantly down-regulated following transfection with miR-6734. To further investigate, we also assessed the effect of miR-6734 on apoptosis in HCT-116 cells. Flow cytometry analysis revealed that miR-6734 concentration-dependently induced apoptosis in HCT-116 cells (Fig 4A and 4B). Moreover, the activation of caspase 3/7, which is a hallmark of apoptosis, was also detected after miR-6734 transfection in HCT-116 cells (Fig 4C).

### The effects of miR-6734 on cell proliferation, cell cycle arrest and apoptosis were abolished by p21 knockdown in HCT-116 cells

**Fig 5:**
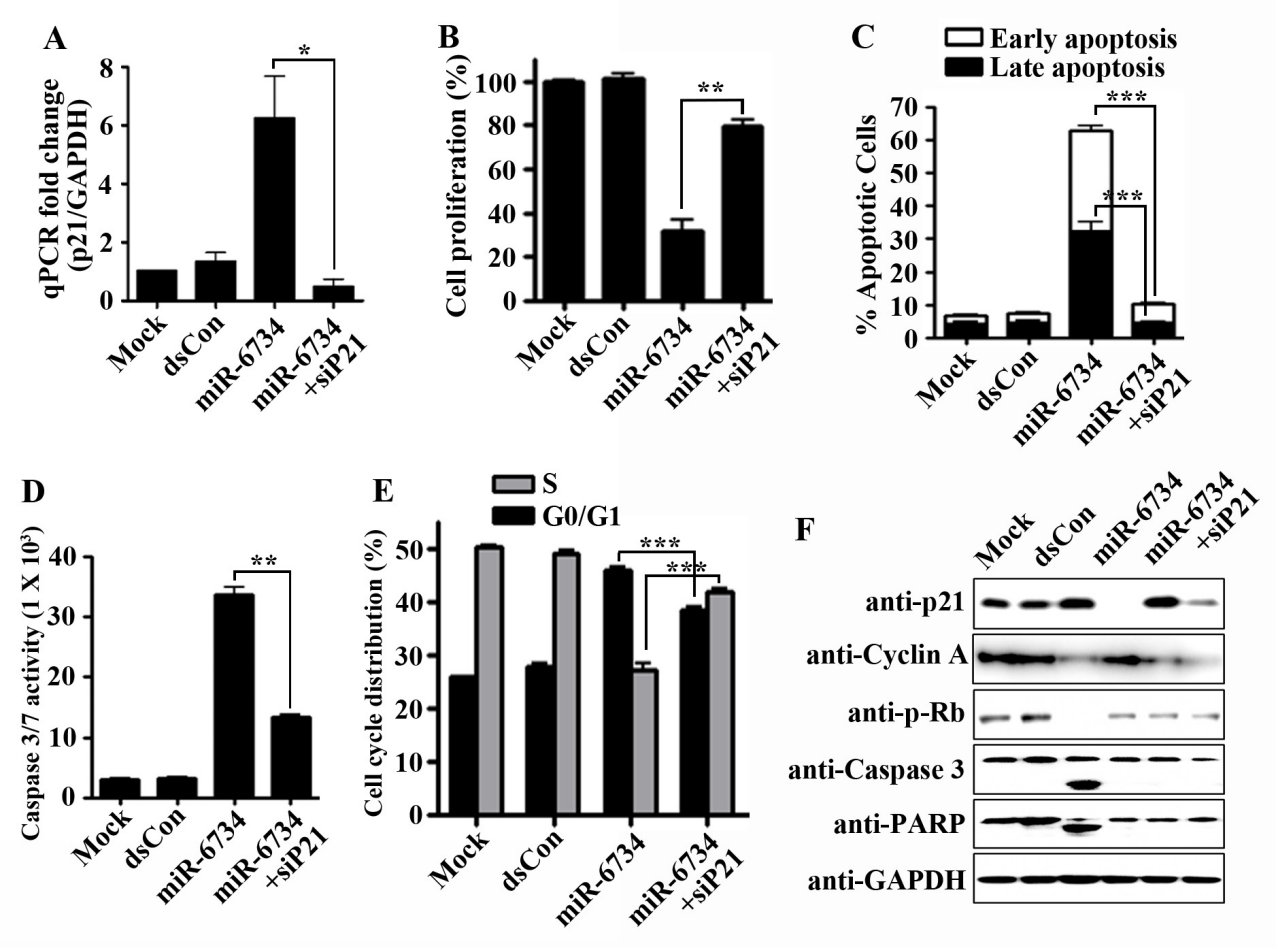
The effects of miR-6734 were abolished by p21 siRNA in HCT-116 cells. Cells were transfected with mock, dsCon or miR-6734 at 10 nmol/L and transfected simultaneously with p21 siRNA (siP21, 10 nmol/L) for p21 knockdown. (A) The mRNA expression of p21 was analyzed by qPCR after 72 h. (B) Viable cells were measured using cell proliferation kit (XTT) after 72 h. (C) The treated cells were stained with propidium iodide and annexin V-FITC after 72 h and apoptotic cells were measured by flow cytometry. The percentage of early and late apoptotic cells were calculated using the WinMDI program. (D) Culture supernatants were collected after 72 h, and the activity of caspase-3/7 was determined via caspase-Glo 3/7 assay kit. (E) Cell cycle analysis was performed after 48 h and the percentage of cells in G0/G1 and S phases were calculated using the ModFit program. Data are presented as mean ± S.D. of triplicate experiments. Statistical significance was analyzed by one-way ANOVA and Dunnett’s t-test (* p < 0.05; ** p < 0.01; *** p < 0.001 versus miR-6734). (F) Protein levels of p21, cyclin-A, p-Rb, caspase 3, PARP and GAPDH in the total cell lysates were determined after 72 h by Western Immunoblot analysis.

To investigate whether the effects of miR-6734 on cell proliferation, cell cycle arrest and apoptosis were mediated by its effect on p21 expression, we analyzed the effects of p21 knockdown on miR-6734-mediated changes in HCT-116 cells. Fig 5A shows that the induction of p21 gene expression by miR-6734 was abrogated by treatment of cells with p21 siRNA. Further study demonstrated that miR-6734-mediated suppression of cell proliferation was reversed by combined transfection with miR-6734 and p21 siRNA in HCT-116 cells (Fig 5B). Fig 5C also shows that the induction of apoptosis by miR-6734 was blocked by p21 siRNA in HCT-116 cells. Accordingly, miR-6734-induced activation of caspase 3/7 activity was also suppressed by p21 knockdown in HCT-116 cells (Fig 5D). Furthermore, transfection of HCT-116 cells with p21 siRNA reversed miR-6734-mediated cell cycle arrest (Fig 5E). Finally, we also confirmed that miR-6734-mediated increases in the expression of p21 and cleavage of caspase 3 and PARP and decreases in the expression of cyclin A and phosphorylated Rb were restored by p21 siRNA (Fig 5F).

### miR-6734 induces histone modification of p21 promoter

**Fig 6:**
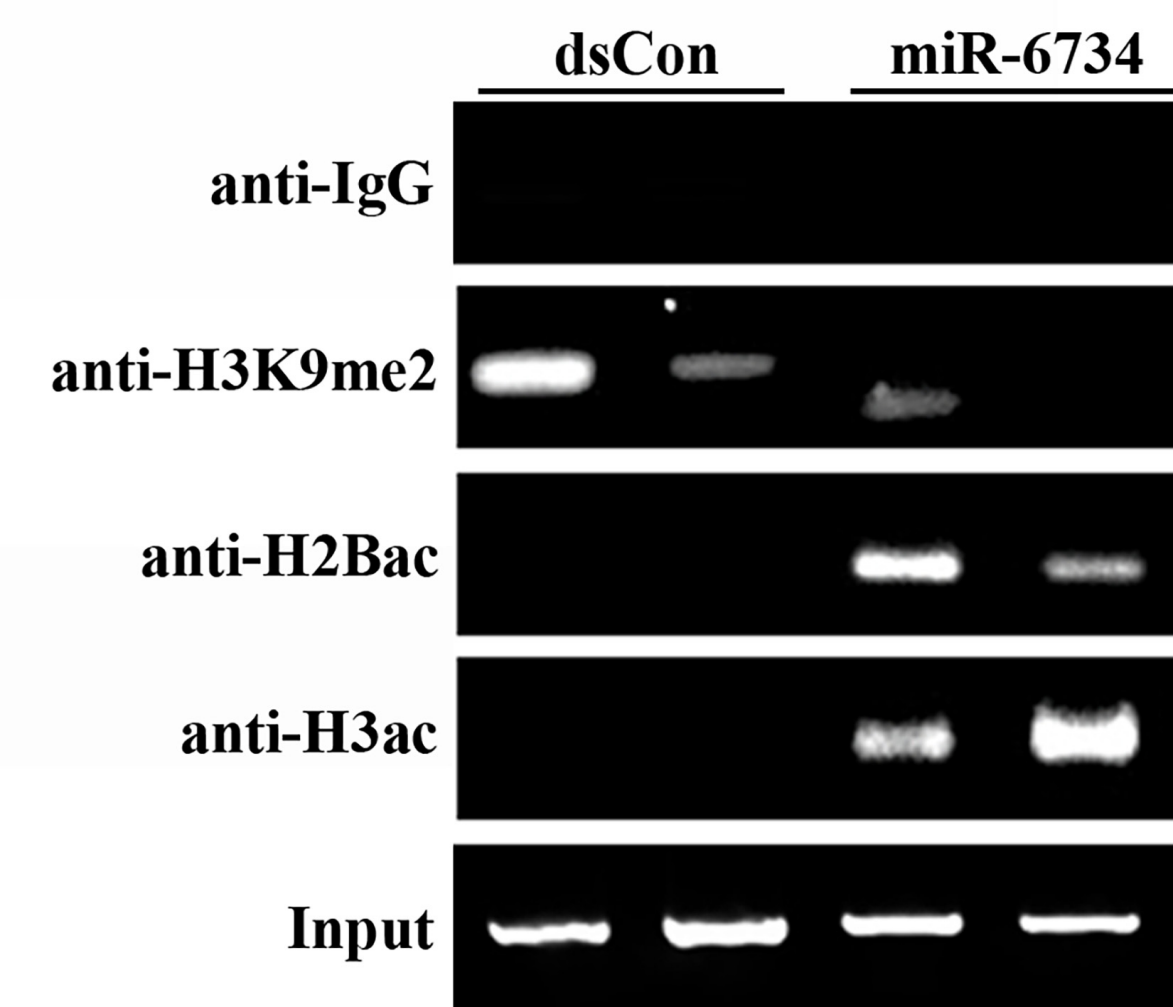
Histone modification of p21 promoter by miR-6734. Chromatin immunoprecipitation (ChIP) assays were performed by using antibodies against IgG, di-methyl-histone-H3-lysine 9 (H3K9me2), acetyl-histone-H2B (H2Bac) and acetyl-histone-H3 (H3ac) to pull down associated DNA. The precipitated DNA was amplified by RT-PCR using primer sets specific to miR-6734 target sites (-360/-260) of p21 gene promoter. Input DNA was amplified as a loading control. DNA pulled down in the IgG antibody served to identify background amplification.

Previous reports demonstrated various types of histone modifications in promoter region following saRNA transfection [16,17]. To investigate whether miR-6734 also induces histone modification of p21 promoter region, we performed ChIP assay using antibodies against modified histones. Fig 6 shows that transfection of cells with miR-6734 decreased a dimethylation of histone H3 lysine 9 (H3K9me2), a well-known marker of transcriptional repression, but increased an acetylation of histone H2B (H2Bac) and histone H3 (H3ac) at miR-6734 target site.

## Discussion

It is becoming evident that miRNAs are playing significant roles in a variety of biological processes, including cell proliferation, apoptosis and differentiation [6]. In the present study, we clearly demonstrated that miR-6734 induces p21 gene expression and suppresses cell proliferation and survival in HCT-116 colon cancer cells. We also showed that miR-6734 induces cell cycle arrest and apoptosis in HCT-116 cells. In addition, our results demonstrated that the kinetics of gene activation and epigenetic modification of promoter region by miR-6734 are similar to those by saRNA. This is the first report showing biological function of miR-6734 and our results suggest that miR-6734 might induce p21 gene expression via a mechanism similar to RNAa.

p21 is a cyclin-dependent kinase inhibitor and plays an important role as a tumor suppressor or as an oncogene depending on the cellular context and circumstances [18]. In case of cell cycle regulation, it has been reported that p21 induces G0/G1 arrest by inhibiting CDK2 activity and G2/M arrest by blocking CDK1 activity [18]. In addition, it was also well-known that the inhibition of CDK2 by p21 dephosphorylates Rb protein, which causes cell cycle arrest via inhibition of E2F1-dependent gene expression [19]. In the present study, we showed that miR-6734 elicited cell cycle arrest at both G0/G1 and G2/M phases in HCT-116 colon cancer cells. We also demonstrated that the phosphorylation of Rb protein was suppressed by miR-6734. However, miR-6734-mediated cell cycle arrest was reversed by knockdown of p21 gene, suggesting that p21 is an important mediator of cell cycle regulation by miR-6734 in HCT-116 cells. p21 is also implicated in the modulation of apoptosis although both anti-apoptotic and pro-apoptotic activity of p21 has been reported [20,21]. Here, we demonstrated that miR-6734 induced apoptosis in HCT-116 cells. Our results also show that the cleavages of caspase 3 and PARP, which were hallmarks of apoptosis induction, was observed after miR-6734 transfection. Moreover, the induction of apoptosis by miR-6734 was attenuated by p21 knockdown in HCT-116 cells. In consistent with our results, previous reports also showed that dsP21-322, a p21 promoter-targeting saRNA, induced apoptosis in hepatocellular carcinoma cells and bladder cancer cells [11–13]. Therefore, these results imply that p21 promoter targeting dsRNAs might promote apoptosis in cancer cells although the exact mechanisms are required to be addressed. Collectively, our results suggest that miR-6734 induces cell cycle arrest and apoptosis via a mechanism dependent on p21.

Li et al. first reported small RNA-mediated gene activation (RNAa) by synthetic dsRNAs targeting gene promoter, which was different from previously well-known small RNA-mediated gene silencing by siRNAs [3]. Further studies revealed that naturally occurring miRNAs can also serve as endogenous mediators of RNAa. It has been reported that miR-373 induced E-cadherin gene expression by binding to its promoter region [7]. Majid et al. also reported that the expression of IL-24 and IL-32 was transcriptionally up-regulated by miR-205 through complementary elements in the gene promoters [22]. miR-744 was also shown to induce cyclin B1 expression by directing histone modification and RNAP II recruitment in a cognate promoter [10]. In contrast to RNAa, recent report by Matsui et al. suggested another mechanism responsible for small RNA-mediated gene activation. In this report, they demonstrated that miR-589 induced the gene expression of cyclooxygenase-2 (COX-2) and phospholipase A2 (PLA2) by binding to COX-2 promoter RNA, a long non-coding RNA which functions as a scaffold for coordinated induction of both COX-2 and PLA2 genes after binding to Ago2/miR-589 complex [23], suggesting a novel mechanism of RNA-mediated gene activation. In the present study, we clearly demonstrated that miR-6734 treatment induces p21 gene expression by binding to p21 promoter region. We also demonstrated that exogenously added inhibitor of miR-6734 down-regulates the basal expression of p21 gene, suggesting an endogenous role of miR-6734 in cancer cells. These results suggest that both exogenous and endogenous miR-6734 up-regulates p21 gene expression by direct binding to p21 promoter.

Up-regulation of gene expression by RNAa has unique kinetics. Induction of gene expression by saRNA is delayed by ~24–48 h compared to knockdown of gene expression by siRNA [5]. In addition, duration of the activity of saRNA is longer than that of siRNA and saRNA-mediated gene induction has been shown to last for nearly 2 weeks [5]. In the present study, it was demonstrated that the induction of p21 gene expression by miR-6734 was evident after 12 days, suggesting that the kinetics of gene expression by miR-6734 follows the kinetics of RNAa. In addition, histone modification has been reported as another characteristic of RNAa. Li et al. reported that H3K9me2 and H3K9me3 were decreased by treatment with saRNA and these histone modifications were associated with RNAa-mediated gene activation [3]. Yang et al. also reported that the activation of prostate apoptosis response-4 (Par-4) by saRNA is accompanied by decrease of H3K9me2 and increase of H3K4me2 [24]. We also demonstrated that miR-6734 decreases dimethylation of H3K9 and increases acetylation of H2B and H3. From these results, it is assumed that miR-6734 induces p21 gene expression in a manner similar to RNAa.

Previous reports also demonstrated targeted activation of p21 promoter by miRNAs. Wang et. al. showed that the level of three miRNAs, including miR-370, miR-1180 and miR-1236, was decreased in bladder cancer tissues and positively correlated with p21 mRNA levels [25]. They also showed that transfection of human bladder cancer cell lines with these three miRNAs induced p21 expression by p21-promoter binding and inhibited the proliferation of cells in p21-dependent manner [25]. Want et. al. also demonstrated that miR-1236 inhibits cell proliferation in human renal cell carcinoma cells by promoter activation [26]. In this report, it has been also demonstrated that the level of both miR-1236 and p21 was significantly down-regulated in the tissues and cell lines of renal cell carcinoma and the combined low expression of both miR-1236 and p21 was an independent poor prognostic factor for poor survival in renal cell carcinoma patients [26]. From these results, it is assumed that the level of endogenous miRNAs was associated with reduced level of p21 in various cancers and targeted activation of p21 by these miRNA might be beneficial for the treatment of cancer patients.

In summary, we demonstrated here that miR-6734 induced p21 gene expression by directly binding to promoter region. Our results also show that miR-6734 induces cell cycle arrest and apoptosis, which is reversed by knockdown of p21 gene. Moreover, we also showed that the induction of p21 gene expression by miR-6734 lasts for about two weeks and is accompanied by chromatin modification. Collectively, these results suggest that selective modulation of p21 gene expression by miR-6734 might be a potential therapeutic option for the treatment of colon cancer.

## Supporting Information

S1 Fig Effects of miR-6734 on cell migration and invasion in HCT-116 cells. (DOCX)

S1 Table dsRNA and primer sequence used in this study. (DOCX)
