# Supplementary material for: miR-6734 Up-Regulates p21 Gene Expression and Induces Cell Cycle Arrest and Apoptosis in Colon Cancer Cells
Source: PLoS One. 2016 Aug 10;11(8):e0160961. doi: 10.1371/journal.pone.0160961 (PMC4979902; doi:10.1371/journal.pone.0160961)

**S1 Fig.** **Effects of miR-6734 on cell migration and invasion in HCT-116 cells.** (A) HCT-116 cells were transfected with Mock, dsCon or the indicated concentrations of miR-6734 and were seeded in the migration dish inserts (Ibidi, Martinsried, Germany). After cells reached to confluency, the inserts were removed and fresh medium was added to start migration. After 24 h, pictures were acquired and image analysis was performed using Image J (NIH image, <http://imagej.net/ImageJ>). (B) HCT-116 cells were transfected with Mock, dsCon or the indicated concentrations of miR-6734 and seeded in the upper insert of a 24-well chamber in BioCoat Matrigel invasion plates (Beckton Dickinson Bioscience, Franlin Lakes, NJ, USA). After 24 h, cells on the upper side of the filters were mechanically removed by scrubbing with a cotton swab and the amount of invaded cells was determined by CellTiter-Glo Luminescent Cell Viability Assay Kit (Promega, Madison, WI, USA). Data are presented as mean ± S.D. of triplicate experiments. Statistical significance was analyzed by one-way ANOVA and Dunnett’s t-test (** p < 0.01; *** p < 0.001 versus mock).


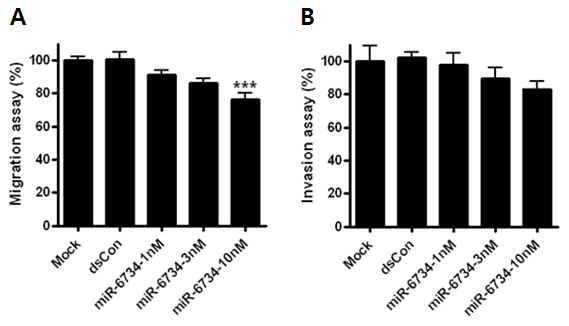

Supplement: S1 Fig — (DOCX) [file pone.0160961.s001.docx]
