# Supplementary material for: miR-6734 Up-Regulates p21 Gene Expression and Induces Cell Cycle Arrest and Apoptosis in Colon Cancer Cells
Source: PLoS One. 2016 Aug 10;11(8):e0160961. doi: 10.1371/journal.pone.0160961 (PMC4979902; doi:10.1371/journal.pone.0160961)

**Supplementary Table 1.** dsRNA and primer sequence

| **dsRNA** | **Sequence (5’-3’)** | |
| --- | --- | --- |
| dsCon | Sense | ACUACUGAGUGACAGUAGA [dT][dT] |
|  | antisense | UCUACUGUCACUCAGUAGU [dT][dT] |
| dsP21-322 | Sense | CCAACUCAUUCUCCAAGUA [dT][dT] |
|  | antisense | UACUUGGAGAAUGAGUUGG [dT][dT] |
| **RT-PCR primer** | **Sequence (5’-3’)** | |
| p21 | Sense | GCCCAGTGGACAGCGAGCAG |
|  | antisense | GCCGGCGTTTGGAGTGGTAGA |
| β-actin | Sense | TGACGGGGTCACCCACACTGTGCCCATCTA |
|  | antisense | CTAGAAGCATTTGCGGTGGACGATGGAGGG |
| GAPDH | Sense | GAAGGTGAAGGTCGGAGTC |
|  | antisense | GAAGATGGTGATGGGATTTC |
| **ChIP primer** | **Sequence (5’-3’)** | |
| p21 (-360/-260) | Sense | GGGGCTCATTCTAACAGTGC |
|  | antisense | GACACATTTCCCCACGAAGT |

**Supplementary Figure 1.** Effects of miR-6734 on cell migration and invasion in HCT-116 cells. HCT-116 cells were transfected with Mock, dsCon or the indicated concentrations of miR-6734. After overnight, cells were used for migration assay and invasion assay. (A)

(B)


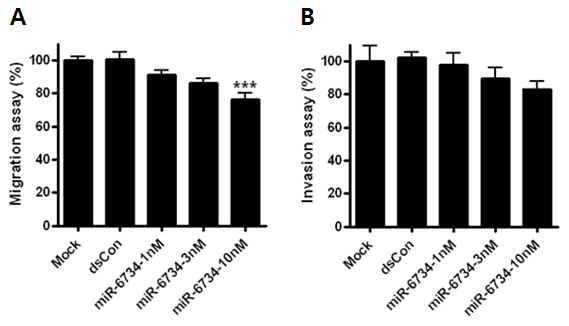

Supplement: S1 Table — (DOCX) [file pone.0160961.s002.docx]
